# The effect of melatonin supplementation on lipid profile, oxidative stress, inflammatory marker, and sleep quality in patients with chronic kidney disease: a GRADE assessed meta-analysis

**DOI:** 10.3389/fnut.2026.1772877

**Published:** 2026-02-06

**Authors:** Qamar Abuhassan, Zahraa Sabah Ghnim, Morug Salih Mahdi, Ashishkumar Kyada, Hamidah Abu Bakar, Neeraj Khare, Dukhbhanjan Singh, Aziz Kubaev, Waam Mohammed Taher, Mariem Alwan, Mahmood Jasem Jawad, Atheer Khdyair Hamad

**Affiliations:** 1 Department of Pharmaceutics and Pharmaceutical Technology, School of Pharmacy, University of Jordan, Amman, Jordan; 2 College of Pharmacy, Alnoor University, Nineveh, Iraq; 3 College of MLT, Ahl Al Bayt University, Karbala, Iraq; 4 Marwadi University Research Center, Department of Pharmaceutical Sciences, Faculty of Health Sciences, Marwadi University, Rajkot, Gujarat, India; 5 Management and Science University, Shah Alam, Selangor, Malaysia; 6 NIMS School of Allied Sciences and Technology, NIMS University, Rajasthan, Jaipur, India; 7 Centre for Research Impact and Outcome, Chitkara University Institute of Engineering and Technology, Chitkara University, Rajpura, Punjab, India; 8 Department of Maxillofacial Surgery, Samarkand State Medical University, Samarkand, Uzbekistan; 9 College of Nursing, National University of Science and Technology, Nasiriyah, Iraq; 10 Pharmacy College, Al-Farahidi University, Baghdad, Iraq; 11 Department of Pharmacy, Al-Zahrawi University College, Karbala, Iraq; 12 Gilgamesh Ahliya University, Baghdad, Iraq

**Keywords:** dyslipidemia, kidney disease, melatonin, meta-analysis, oxidative stress, sleep

## Abstract

**Background:**

Melatonin (MLT) might benefit heart and metabolic health, as well as sleep quality, in individuals with chronic kidney disease (CKD), but the research findings are mixed. To better understand this, we conducted a systematic review and meta-analysis of randomized controlled trials (RCTs) to assess how MLT affects people with CKD.

**Methods:**

Scopus, the Cochrane Library, PubMed, Web of Science, and Embase were searched up to May 30, 2025, for RCTs reporting lipid profiles, oxidative stress markers, or sleep quality. Random-effects meta-analyses were performed, and results are presented as weighted mean differences (WMDs) with 95% confidence intervals (CIs).

**Results:**

Ten RCTs (12 trials) were included. MLT supplementation significantly increased high-density lipoprotein cholesterol (HDL-C: WMD = 1.87 mg/dL, 95% CI: 0.24, 3.50, *p* = 0.025; *I*^2^ = 38.9, *p* = 0.179), and reduced malondialdehyde (MDA: −1.28 μmol/L, 95% CI: −2.50, −0.06, *p* = 0.039; *I*^2^ = 93.4, *p* < 0.001), and improved sleep quality (PSQI: WMD = -3.75, 95% CI: −6.92, −0.57, *p* = 0.021; *I*^2^ = 94.2, *p* < 0.001). Also, MLT supplementation had no significant effect on triglycerides, total cholesterol, low-density lipoprotein cholesterol, or C-reactive protein.

**Conclusion:**

Supplementing with MLT in CKD can gently raise HDL-C levels, decrease oxidative stress, and improve sleep quality. While these effects are encouraging, more extensive and carefully planned clinical trials are necessary to verify the actual benefits.

## Introduction

Chronic kidney disease (CKD) is a major global health issue, affecting roughly 10–15% of adults worldwide (1). Its incidence is rising due to aging populations and the growing prevalence of diabetes, hypertension, and obesity (2). CKD causes a gradual decline in kidney function and carries a much higher risk of cardiovascular disease (CVD). In fact, CVD is the leading cause of illness and death in CKD patients.

Key factors that raise cardiovascular risk and promote CKD progression are dyslipidemia, persistent inflammation, and oxidative stress (3). People with CKD often have abnormal blood lipid levels, high triglycerides (TG), low HDL cholesterol, and altered LDL particles, which contribute to atherosclerosis (4). They also have chronic inflammation [with elevated cytokines such as interleukin-6 (IL-6) and tumor necrosis factor-*α* (TNF-α)] and increased oxidative stress (with increased reactive oxygen species and reduced antioxidant defenses), which impair blood vessel function, worsen kidney damage, and harm overall health (5). Many CKD patients also experience sleep disturbances such as insomnia and poor sleep quality, which further harm their health and quality of life (6).

Melatonin (MLT) is a hormone secreted by the pineal gland that helps regulate the circadian rhythm and the sleep–wake cycle. In addition to helping regulate sleep, melatonin also functions as an antioxidant, has anti-inflammatory effects, and offers protective benefits for the heart (7, 8). Because of these qualities, MLT shows potential for treating several problems linked to CKD (9).

Some studies suggest that MLT supplements might help improve sleep quality and support metabolic health in people with CKD (10). For instance, MLT could affect blood lipid and glucose levels (11). However, the findings are inconsistent and not conclusive (12–16), partly because of variations in study methods, doses, treatment lengths, and patient differences. So, a thorough systematic review and meta-analysis are necessary to compile all the existing data and better understand how melatonin might play a role in managing CKD.

Despite numerous clinical trials of MLT in CKD, there remains no consensus on its effectiveness. Without a comprehensive review of the evidence, clinicians lack clear guidance. This meta-analysis aims to systematically synthesize evidence from randomized controlled trials (RCTs) and observational studies to evaluate the effects of MLT supplementation on metabolic factors (e.g., blood lipids, glycemic, inflammatory markers, and oxidative stress markers) and sleep quality in patients with CKD. The goal is to clarify the potential benefits of melatonin for this high-risk group and to guide future research and clinical practice.

## Methods

This systematic review was conducted according to the PRISMA (Preferred Reporting Items for Systematic Reviews and Meta-Analyses) 2020 guidelines (17).

### Search strategy

A systematic literature searches of online databases, including Scopus, the Cochrane Library, PubMed, Web of Science, and Embase, was conducted through May 30, 2025, with no restrictions on geographic area, publication date, or language. RCTs evaluating the effects of MLT on patients with CKD were managed using EndNote. The search terms used in this query include the following combinations (Supplementary Table 1). To ensure a comprehensive search, a back- and front-reference check was conducted on the identified articles to identify any studies that might have been overlooked in the primary systematic search.

### Inclusion and exclusion criteria

The inclusion criteria were: (1) studies, (2) reported results on lipid profiles, oxidative stress (MDA), inflammatory markers (CRP), (3) and sleep quality (PSQI) (4) the intervention group received an MLT supplement, while the control group received a placebo or no treatment in patients diagnosed with CKD. The exclusion criteria were: (1) meta-analyses, comments, letters, reviews, case reports, animal studies, *in vivo* and *in vitro*, (2) insufficient reporting of data, and (3) co-supplementation of data. We have excluded observational, case–control, cohort, cross-sectional, retrospective, and environmental studies. Studies without our variables of interest, laboratory studies, studies with insufficient data, and those in which CD was not administered orally were also excluded.

### Study selection and data extraction

Data selection and extraction were conducted independently by two researchers, using inclusion and exclusion criteria, based on a full-text review of the articles. Initially, the titles and abstracts of all the articles we found were reviewed to find studies that might be related. Those that seemed relevant were then chosen for a detailed full-text review. During this detailed review, two reviewers independently examined each study to decide whether it should be included. Any conflicting decisions by the evaluators to include a study were resolved by consensus. The following data have been extracted from the pooled data: (1) reference (year of publication and name of first author); (2) country of study; (3) characteristics of the population (age, sex), and size of the sample. (4) the characteristics of the intervention, the dose of the adjuvant, placebo, or control, and the type of RCT (parallel, or crossover).

### Quality assessment and GRADE approach

The methodological quality of the RCTs was evaluated by two independent authors using the Cochrane Risk of Bias (ROB 2) tool (18), who assessed randomization, allocation, blinding, incomplete data, and selective reporting; a third author resolved the inconsistencies.

The GRADE methodology was used to evaluate the overall quality of the evidence (19). This method considers factors such as risk of bias, accuracy, inconsistency, and publication bias to assess the reliability of the evidence.

### Statistical analysis

Synthesis data consisted of mean changes and corresponding SDs, with the DerSimonian-Laird random-effects model implemented in Stata 17.0 (Stata Corp., College Station, Texas, United States). The effect estimates were presented as weighted average differences (WMDs) with 95% confidence intervals (CIs). Crossover trials were analyzed using end-of-period data, and in multi-arm studies the control group was split or a single intervention arm was included to avoid double-counting, following Cochrane Handbook guidance. Inter-study heterogeneity was assessed using the Cochran Q test and the *I*^2^ statistic; *p*-values < 0.1 or *I*^2^ > 50% indicated significant inter-study heterogeneity. Sensitivity analyses were performed to investigate the robustness of the findings. In addition, subgroup analyses were conducted to identify sources of differences in dose, age, and treatment duration. Possible evidence of publication bias was statistically assessed using Begg’s test across the RCTs (20).

## Results

### Literature screening and study characteristics

**Figure 1:**
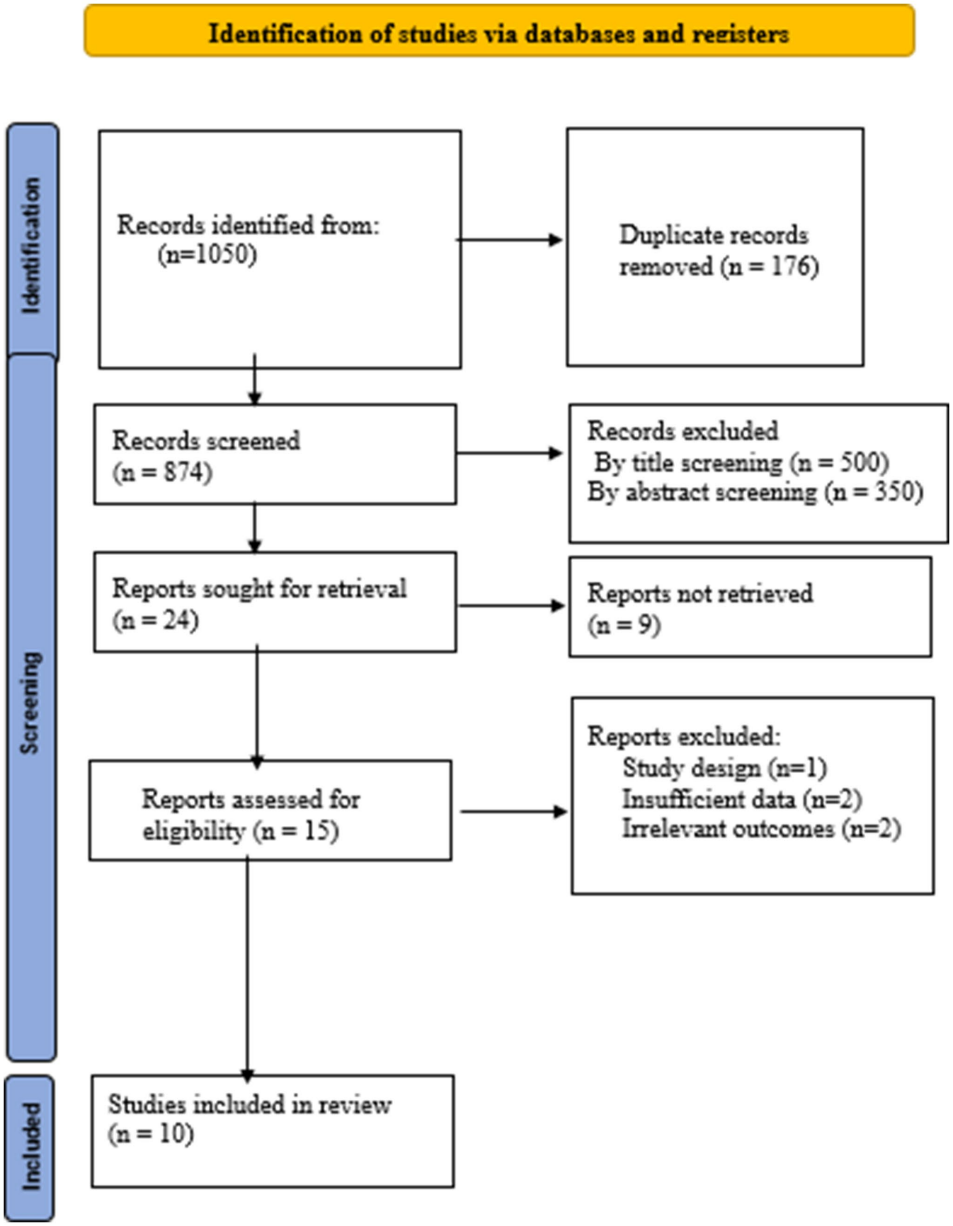
PRISMA flow diagram of study selection.

Figure 1 shows the process of selecting studies. In total, 1,050 articles were subject to the first review. After removing duplicates, the authors reviewed the titles and abstracts of the 874remaining datasets, excluding 750 irrelevant links. In the next step, 14 documents were excluded following a full-text examination. Finally, 10 eligible RCTs with 12 arms were included in this meta-analysis. As shown in Table 1, trials were conducted between 2013 (12) and 2025 (15), with sample sizes ranging from 13 to 102 participants, treatment durations of 4 to 24 weeks, and MLT supplement doses of 3 to 10 mg/day. The trials were conducted in different countries, including Iran, Africa, and Iraq.

**Table 1:** Characteristics of included studies in the meta-analysis.

Authors, Location, year	Study Design	Participants, n	Mean Age (Int/ Con)	Health condition	Intervention	Duration (Week)	Control	Outcomes
Sadeghi, Iran. 2025 (15)	DB, RCT	M/F: 41	64/65	Diabetic with CKD	10 mg/day of melatonin	10	Placebo	CRP and MDA
Ostadmohammadi, Iran. 2019 (23)	DB, RCT	M/F: 53	65/64	Diabetic with HD	10 mg/day of melatonin	12	Placebo	TG, TC, LDL-C, HDL-C, CRP, MDA, and PSQI
Al Lami, Iraq. 2018 (22)	SB, RCT	M/F: 41	58/56	CKD	5 mg/day of melatonin	12	Placebo	TG, TC, LDL-C, HDL-C, and MDA
Marzougui, Africa.2024 (33)	DB, RCT	M/F: 22	49/49	HD	Patients treated with Exercise plus 3 mg melatonin	12	Exercise plus Placebo	CRP and MDA
Jafari, Iran.2023 (13)	RCT	M/F: 102	58/58	HD	3 mg/day of melatonin	6	Placebo	PSQI
Talari, Iran.2022 (34)	DB, RCT	M/F: 32	40–85	DN	10 mg/day of melatonin	24	Placebo	CRP
Zahed, Iran.2022 (16)	CT	M/F: 23	60	HD	3 mg/day of melatonin	12	Not reported	TG, TC, HDL-C, and CRP
Edalat Nejad, Iran.2013 (12)	DB, RCT	M/F: 68	58	HD	3 mg/day of melatonin	6	Placebo	TG, TC, LDL-C, HDL-C, and PSQI
Marzougui, Africa.2021 (21)	DB, RCT	M/F: 13	43	HD	Exercise plus 3 mg/day melatonin Control plus 3 mg/day melatonin	4	Exercise plus placebo Control plus placebo	CRP
Marzougui, Africa.2023 (14)	DB, RCT	M/F: 13	43	HD	Exercise plus 3 mg/day melatonin Control plus 3 mg/day melatonin	4	Exercise plus placebo Control plus placebo	MDA

DB, RCT; double-blinded randomized controlled trial: SB, RCT; single-blinded randomized controlled trial: CT; Clinical trial, M; Male: F; Female: CKD; chronic kidney disease: DN, diabetic nephropathy: HD; Hemodialysis.

### Risk of bias and certainty of evidence

Table 2 presents the methodological quality assessment of the trials using the RoB2 tool, reflecting the authors’ judgments across the risk-of-bias domains. The certainty of evidence was high for HDL-C and moderate for all other outcomes, including TC, TG, LDL-C, MDA, CRP, and PSQI (Supplementary Table 2).

**Table 2:** Quality assessment.

Study	Randomization process	Deviations from intended interventions	Missing outcome data	Measurement of outcomes	Selection of reported results	Overall risk of bias
Sadeghi et al. (15)	Low risk	Low risk	Low risk	Low risk	Some concerns	Some concerns
Ostadmohammadi et al. (23)	Low risk	Low risk	Low risk	Low risk	Low risk	Low risk
Al Lami (22)	Some concerns	Low risk	Low risk	Low risk	Some concerns	Some concerns
Marzougui et al. (33)	Some concerns	Low risk	Low risk	Low risk	Some concerns	Some concerns
Jafari et al. (13)	Low risk	Low risk	Low risk	Low risk	Low risk	Low risk
Talari et al. (34)	Some concerns	Low risk	Some concerns	Low risk	Some concerns	Some concerns
Zahed et al. (16)	Low risk	Low risk	Low risk	Low risk	Low risk	Low risk
Edalat-Nejad et al. (12)	Some concerns	Low risk	Low risk	Low risk	Some concerns	Some concerns
Marzougui et al. (21)	Some concerns	Low risk	Low risk	Low risk	Some concerns	Some concerns
Marzougui et al. (14)	Some concerns	Low risk	Low risk	Low risk	Some concerns	Some concerns

### Effects of MLT on lipid profile

**Figure 2:**
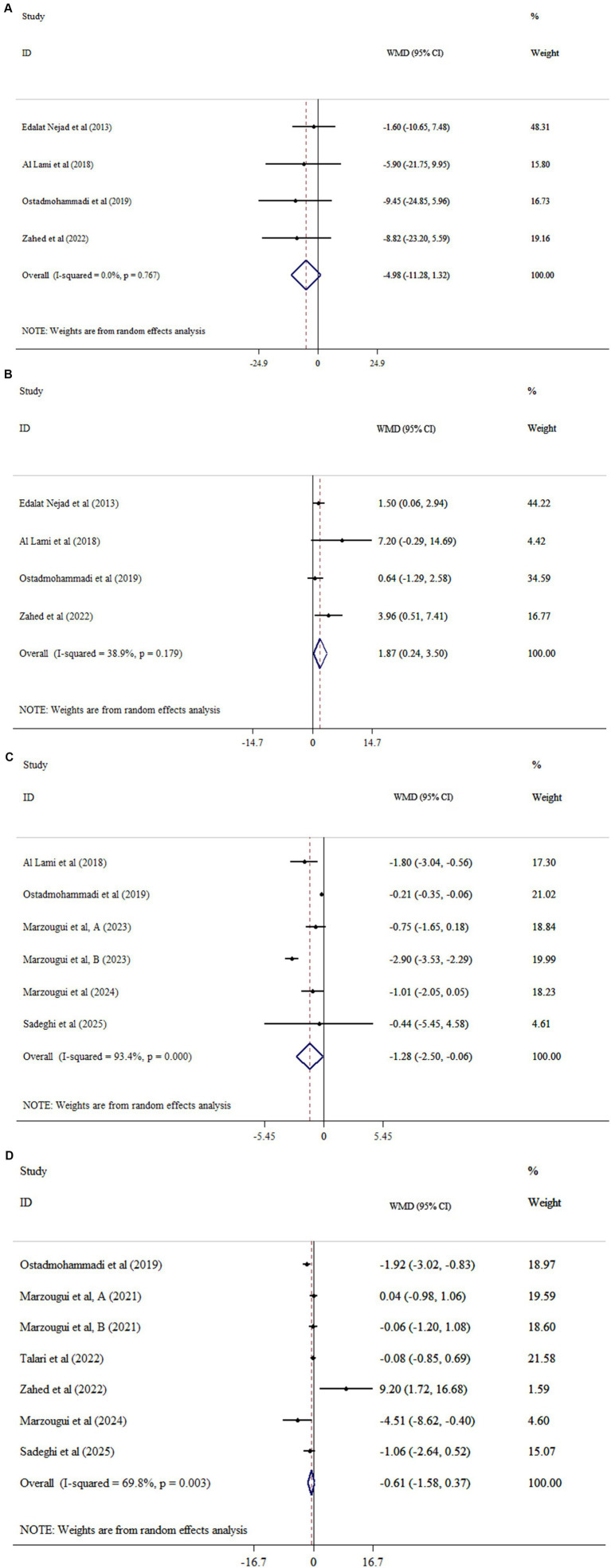
Forest plots of the effects of melatonin (MLT) on **(A)** TC, **(B)** TG, **(C)** LDL-C, and **(D)** HDL-C.

The results of our analysis showed that MLT did not significantly affect TC (WMD = −4.98, 95% CI: −11.28, 12.81, *p* = 0.122; *I*^2^ = 0.0%, *p* = 0.767; Figure 2A), TG (WMD = −16.95, 95% CI: 38.68, 4.77, *p* = 0.126; *I*^2^ = 69.1%, *p* = 0.021; Figure 2B), or LDL-C (WMD = −4.95, 95% CI: −12.11, 2.21, *p* = 0.175; *I*^2^ = 24.9%, *p* = 0.264; Figure 2C) compared with placebo. In addition, meta-analysis demonstrated that MLT supplementation significantly increased HDL-C (WMD = 1.87, 95% CI: 0.24, 3.50, *p* = 0.025; *I*^2^ = 38.9, *p* = 0.179; Figure 2D). Age, dosage, and intervention duration were identified as sources of heterogeneity. However, subgroup analyses indicated that the results remained statistically no significant (Table 2).

### Effects of MLT on oxidative stress and inflammation

**Figure 3:**
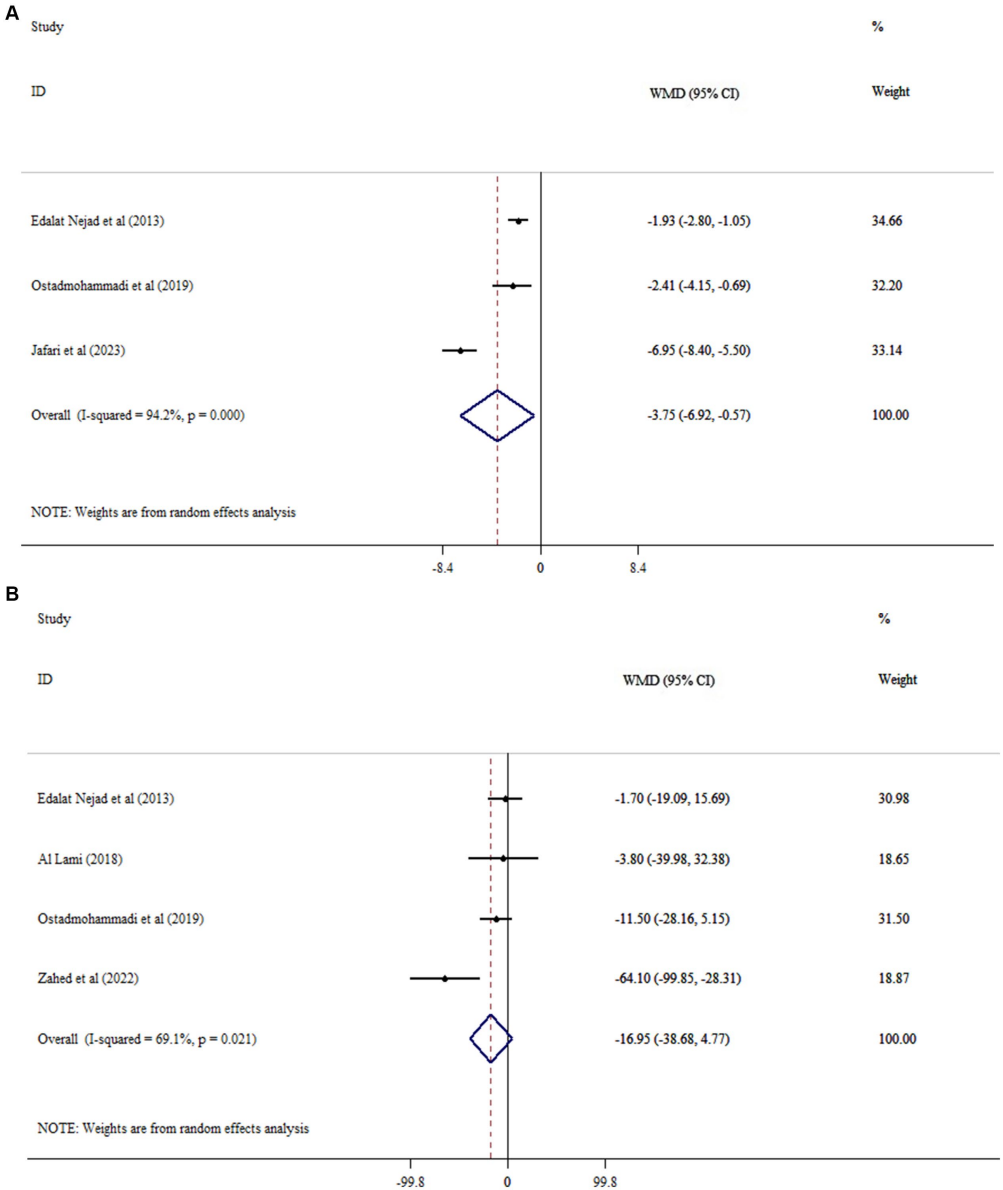
Forest plots of the effects of melatonin (MLT) on **(A)** MDA and **(B)** CRP.

Meta-analysis demonstrated that MLT supplementation significantly reduced MDA (WMD = −1.28, 95% CI: −2.50, −0.06, *p* = 0.039; *I*^2^ = 93.4, *p* < 0.001; Figure 3A), whereas CRP was not significantly affected (WMD = −0.61, 95% CI: −1.58, 0.37, *p* = 0.224; *I*^2^ = 69.8, *p* = 0.003; Figure 3B) compared to placebo. Subgroup analysis showed that, compared with placebo, MLT supplementation was associated with a more robust decrease in MDA levels at doses < 5 mg/day and in participants <60 years old (Table 3).

**Table 3:** Subgroup analysis based on duration, dose, and age.

Outcomes	Number of trials	WMD (95% CI)
Triglycerides		
Age		
<60	2	−2.09 (−17.77, 13.58)
≥60	2	−35.32 (−86.64, 16.00)
Intervention duration (week)		
<12	1	−1.70 (−19.09, 15.69)
≥12	3	−24.81 (−57.45, 7.83)
Dosage (mg/day)		
<5 of supplementation	2	−30.86 (−91.88, 30.16)
≥5 of supplementation	2	−10.15 (−25.28, 4.98)
Total cholesterol		
Age		
<60	2	−2.66 (−10.53, 5.21)
≥60	2	−9.11 (−19.63, 1.40)
Intervention duration (week)		
<12	2	−1.60 (−10.66, 7.46)
≥12	2	−8.13 (−16.89, 0.63)
Dosage (mg/day)		
<5 of supplementation	2	−3.65 (−11.32, 4.02)
≥5 of supplementation	2	−7.73 (−18.77, 3.32)
High-density lipoprotein cholesterol		
Age		
<60	2	3.12 (−1.92, 8.15)
≥60	2	1.98 (−1.21, 5.17)
Intervention duration (week)		
<12	1	1.50 (0.06, 2.94)
≥12	3	2.80 (−0.51, 6.11)
Dosage (mg/day)		
<5 of supplementation	2	2.21 (0.03, 4.39)
≥5 of supplementation	2	2.88 (−3.22, 8.98)
C-reactive protein		
Age		
<60	4	−0.17 (−0.89, 0.55)
≥60	3	−0.53 (−3.02, 1.96)
Intervention duration (week)		
<12	3	−0.20 (−0.89, 0.48)
≥12	4	−0.85 (−3.03, 1.33)
Dosage (mg/day)		
<5 of supplementation	4	−0.15 (−2.03, 1.74)
≥5 of supplementation	3	−0.97 (−2.20, 0.27)
Malondialdehyde		
Age		
<60	4	−1.65 (−2.80, −0.49)
≥60	2	−0.21 (−0.36, −0.07)
Intervention duration (week)		
<12	3	−1.70 (−3.61, 0.22)
≥12	3	−0.86 (−1.82, 0.11)
Dosage (mg/day)		
<5 of supplementation	3	−1.59 (−3.09, −0.09)
≥5 of supplementation	3	−0.82 (−2.15, 0.50)

### Effects of MLT on sleep quality

**Figure 4:**
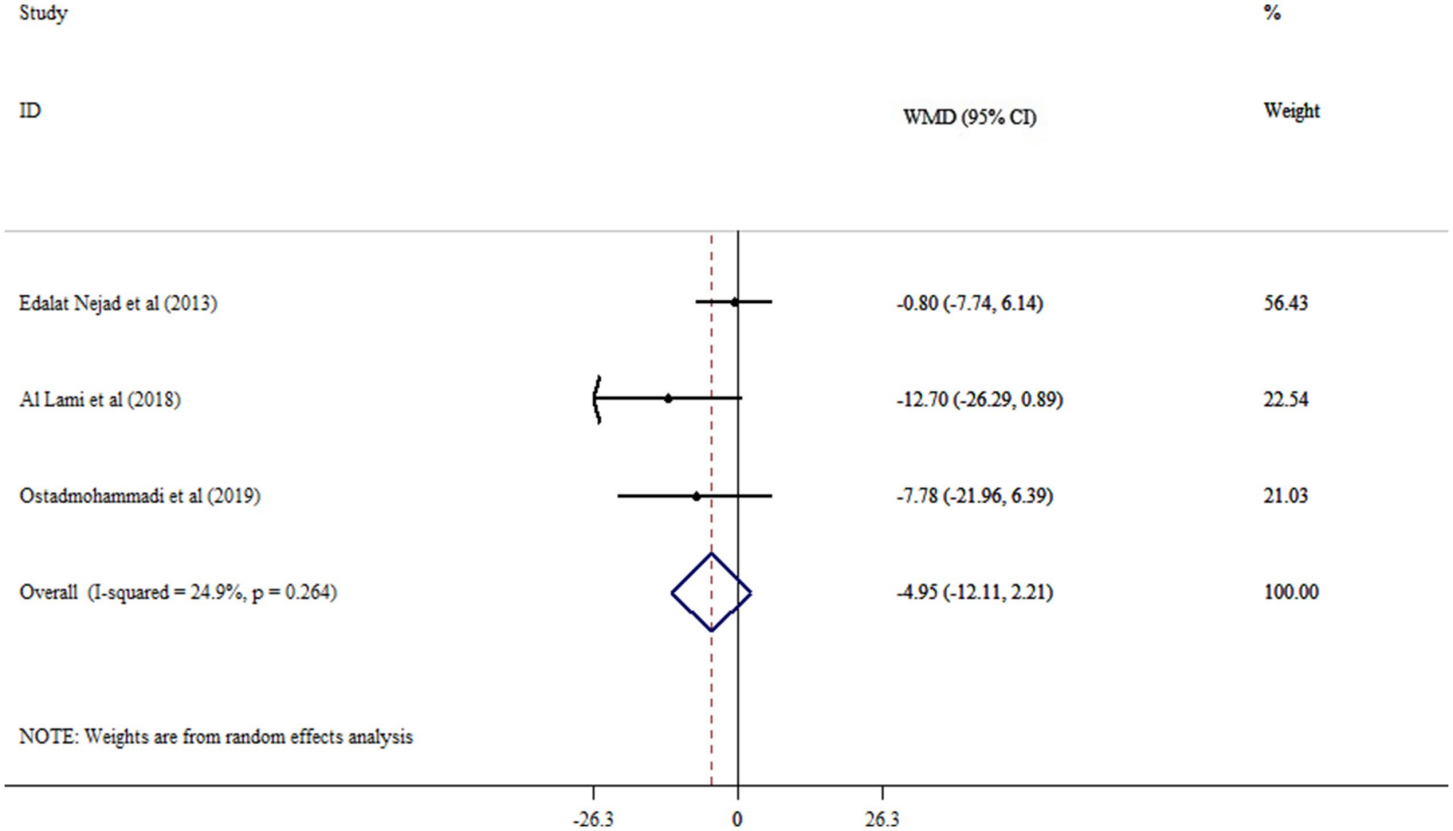
Forest plot of the effect of melatonin (MLT) on sleep quality (PSQI).

The result of combining the data showed a significant effect of the MLT supplementation on Pittsburgh Sleep Quality Index (PSQI) (WMD = -3.75, 95% CI: −6.92, −0.57, *p* = 0.021; *I*^2^ = 94.2, *p* < 0.001; Figure 4).

### Sensitivity analysis and publication bias

Sensitivity analysis showed no significant effect on the TG, TC, and LDL-C and confirmed the robustness of these results. However, for the HDL-C (12, 16), MDA (21, 22), and PSQI (23), the pooled effects changed from statistically significant to non-significant after the exclusion of individual studies (Supplementary Figures 1–7). According to Begg’s tests, there was no evidence of significant publication bias for TG (*p* = 0.999), TC (*p* = 0.734), LDL-C (*p* = 0.602), HDL-C (*p* = 0.174), CRP (*p* = 0.764), MDA (*p* = 0.999), and PSQI (*p* = 0.999).

## Discussion

This meta-analysis of RCTs involving patients with CKD shows that MLT supplementation offers some small benefits for certain outcomes. However, many lipid and inflammatory markers did not show significant changes. Notably, MLT was linked to an increase in HDL-C, a decrease in MDA, and better sleep quality. On the other hand, levels of TC, LDL-C, TG, and CRP did not significantly alter. In the following discussion, we examine what these findings mean clinically, explore possible reasons for differences across studies, and consider what this means for future research.

### Effects on lipid profiles

MLT supplementation led to a small but statistically significant increase in HDL-C (mean difference approximately +1.9 mg/dL) (24). HDL particles exert anti-atherogenic effects through reverse cholesterol transport as well as antioxidant and anti-inflammatory mechanisms, making this finding potentially relevant in CKD, a population in which low HDL-C levels are common (25). The observed increase in HDL-C was modest in scale. Importantly, medications aimed at raising HDL-C levels have generally not led to a decrease in cardiovascular events (26). This suggests that while melatonin may slightly raise HDL-C, the clinical significance of this change is uncertain. An increase of about 2 mg/dL alone is unlikely to provide meaningful cardiovascular benefit unless it is accompanied by improvements in HDL function or other positive changes in lipid profiles. MLT supplementation did not significantly affect TC, LDL-C, or TG levels in the pooled analysis. This result aligns with several individual studies that found no significant changes in LDL-C or triglyceride levels following short-term supplementation (27). The lack of effect could be due to methodological issues, such as small sample sizes, brief intervention periods, or interference from other lipid-lowering treatments, since many CKD patients are on medications like statins that strongly affect lipid profiles.

It is also conceivable that higher doses or longer durations of MLT treatment may be necessary to observe lipid-modulating effects beyond those investigated in the current studies (28). Considering the considerable variability among the trials, including differences in CKD stages, dialysis status, baseline lipid levels, and concomitant medications, these null results should be interpreted with caution. They do not rule out potential benefits for certain CKD subpopulations, underscoring the importance of conducting larger, well-designed studies with prolonged follow-up periods.

### Effects on oxidative stress and inflammation

Inflammatory outcomes in the present meta-analysis should be interpreted in the context of the limited scope of the included studies. Only CRP was consistently reported across the eligible RCTs, which restricted our ability to evaluate the broader anti-inflammatory effects of MLT. MLT supplementation markedly decreased MDA levels, with a weighted mean difference of approximately −1.3 units and a *p*-value near 0.04. MDA is a recognized biomarker for lipid peroxidation and oxidative stress. A reduction in MDA levels indicates that melatonin may exert antioxidant effects in patients with CKD, consistent with its established role as a free-radical scavenger (15). Although theoretically, lowering MDA could safeguard cells from oxidative damage, the magnitude of change observed was modest, similar to the change in HDL-C, and it remains uncertain whether this slight decrease translates into significant clinical benefits.

MLT’s effects on MDA may also be context-dependent. In other experimental models, such as trauma, the timing of administration influenced whether MDA levels increased or decreased (29). In the trials included in this meta-analysis, no reduction in CRP, a general marker of systemic inflammation, was observed. This suggests that short-term MLT supplementation does not significantly affect inflammatory status in CKD patients. Likewise, high-sensitivity CRP (hs-CRP) levels remained stable. Due to the complex and multifactorial nature of CKD-related inflammation, more effective or longer-lasting interventions are generally needed to achieve noticeable decreases. The absence of a CRP-lowering effect indicates that MLT’s anti-inflammatory actions, such as inhibiting NF-κB, were not enough to alter circulating inflammatory markers in these studies (30). Overall, our findings suggest that melatonin more reliably reduces oxidative stress, as shown by MDA levels, than it does reduce systemic inflammation contexts.

### Effects on sleep quality

Melatonin supplementation produced a meaningful improvement in sleep, as reflected by pooled Pittsburgh Sleep Quality Index (PSQI) scores. Specifically, global PSQI scores decreased by approximately 3.8 points, indicating improved subjective sleep quality. This aligns with melatonin’s established role as a circadian regulator: exogenous melatonin promotes sleep onset and reduces sleep latency in humans.

Patients with CKD often have disrupted melatonin rhythms, partly because their bodies cannot clear it properly (31, 32). This disruption can lead to difficulties falling asleep and poor sleep quality. Research has shown that in dialysis patients, taking melatonin supplements can improve how long and how well they sleep. Better sleep can enhance overall quality of life and may also have positive effects on heart and metabolic health.

### Clinical significance of changes

While the observed changes in HDL-C and MDA were statistically significant, their small size means we should be cautious in interpreting these results. A roughly 2 mg/dL increase in HDL-C is much less than what is typically seen with standard lipid-lowering therapies, and simply raising HDL levels has not consistently improved heart health outcomes. Similarly, a reduction of approximately 1.3 units in MDA suggests decreased lipid peroxidation, but it is unclear if this leads to significant reductions in tissue oxidative damage. While MDA is a widely used marker for oxidative stress, it does not directly induce disease.

In simpler terms, our findings indicate initial biochemical effects, such as a slight rise in HDL and a reduction in MDA, but these alone may not translate into significant clinical benefits. This underscores the distinction between statistical significance and genuine health improvements. To determine whether these minor biomarker shifts impact patient health, larger, more rigorous studies that examine real health outcomes like heart attacks or kidney disease progression are essential.

### Limitations and heterogeneity

Our findings should be approached with caution because of several limitations. Firstly, the trials involved different patient groups, some with dialysis and others without, varied MLT doses ranging from 3 to 10 mg per day, and different durations from 4 to 24 weeks, which created clinical and statistical differences. Secondly, only 10 RCTs with 12 study arms were included, most having small sample sizes of fewer than 50 participants. This limits the accuracy of the results and reduces the power of subgroup analyses. Sensitivity tests indicated that removing individual studies might cause some significant findings, such as effects on HDL-C, MDA, and PSQI, to disappear. Thirdly, the follow-up periods were generally short, making it impossible to evaluate long-term outcomes like cardiovascular events or the progression of CKD. Lastly, biases could still be present, including unreported small negative studies and the absence of individual patient data to adjust for baseline differences like statin use or CKD severity.

### Recommendations for future research

To fill current gaps in research, future studies should focus on conducting adequately powered trials to confirm these results and assess clinical outcomes such as cardiovascular events, progression of CKD, and mortality, in addition to biochemical markers. It’s also worth investigating whether varying doses or the timing of administration, like evening versus morning, can enhance lipid profiles, antioxidant effects, or sleep quality. Evaluating HDL particle function, including cholesterol efflux and anti-inflammatory capacity, along with a broader range of oxidative stress markers, will help us understand the full antioxidant potential of MLT. Studies should be tailored specifically for dialysis and non-dialysis patients, as well as for older or more advanced CKD groups, to better identify who benefits the most. It’s also important to use the same methods consistently to measure lipid levels, oxidative stress markers, and sleep quality, like the Pittsburgh Sleep Quality Index, to minimize variability and make it easier to compare results across different studies.

## Conclusion

We concluded that although MLT supplements increase HDL-C levels, they reduce oxidative stress, as indicated by MDA, and improve sleep quality in CKD patients. However, they did not affect other lipid or inflammatory markers. The clinical significance of these modest benefits is uncertain due to variability across studies and the small sample sizes. These findings support the need for larger, carefully designed studies to assess whether melatonin can benefit CKD patients.

## Data Availability

The datasets presented in this study can be found in online repositories. The names of the repository/repositories and accession number(s) can be found in the article/Supplementary material.
