# Supplementary material for: The effect of melatonin supplementation on lipid profile, oxidative stress, inflammatory marker, and sleep quality in patients with chronic kidney disease: a GRADE assessed meta-analysis
Source: Front Nutr. 2026 Feb 6;13:1772877. doi: 10.3389/fnut.2026.1772877 (PMC12920205; doi:10.3389/fnut.2026.1772877)
Supplement: Supplementary file 1 [file Table_1.docx]

**Supplementary Table 1**. Search strategy

("chronic kidney disease (CKD) "OR "chronic renal failure" OR "end stage renal failure (ESRD) " OR "hemodialysis(HD)"), intervention ("melatonin" AND "use" OR "supplementation" OR "intake"), and outcomes ("triglycerides (TG)" OR "total-cholesterol (TC)" OR "LDL-cholesterol (LDL)" OR "low-density lipoprotein "OR "HDL-cholesterol" OR "High-density lipoprotein "OR "inflammatory markers" OR "inflammatory biomarkers" OR "C-reactive protein (CRP)" OR "malondialdehyde (MDA)" OR "oxidative stress biomarkers" OR "oxidative stress markers" OR "sleep quality" OR "Pittsburgh Sleep Quality Index(PSQI)").

**Supplementary Table 2:** GRADE profile.

| **Certainty assessment** | | | | | | | **Certainty** |
| --- | --- | --- | --- | --- | --- | --- | --- |
| **№ of studies** | **Study design** | **Risk of bias** | **Inconsistency** | **Indirectness** | **Imprecision** | **Publication bias** |  |
| TC | RCTs | Not Serious | Not Serious | Not Serious | Serious | Not Serious | Moderate |
| TG | RCTs | Not Serious | Not Serious | Not Serious | Not Serious | Not Serious | Moderate |
| LDL-C | RCTs | Not Serious | Not Serious | Not Serious | Serious | Not Serious | Moderate |
| HDL-C | RCTs | Not Serious | Not Serious | Not Serious | Not Serious | Not Serious | High |
| MDA | RCTs | Not Serious | Serious | Not Serious | Not Serious | Not Serious | Moderate |
| CRP | RCTs | Not Serious | Not Serious | Not Serious | Serious | Not Serious | Moderate |
| PSQI | RCTs | Not Serious | Serious | Not Serious | Not Serious | Not Serious | Moderate |

**Supplementary figure 1. TC**

**Supplementary figure 2. TG**

**Supplementary figure 3. LDL-C**

**Supplementary figure 4. HDL-C**

**Supplementary figure 5.** CRP

**Supplementary figure 6. MDA**

**Supplementary figure 7. PSQI**
